# Covalent linkage of bacterial voltage-gated sodium channels

**DOI:** 10.1186/s13628-019-0049-5

**Published:** 2019-04-27

**Authors:** Huaping Sun, Zeyu Zheng, Olena A. Fedorenko, Stephen K. Roberts

**Affiliations:** 1 Division of Biomedical and Life Sciences, Faculty of Health and Medicine, Lancaster University, Lancaster, LA1 4YQ UK; 2 Present Address: School of Life Sciences, University of Nottingham, Nottingham, NG7 2UH UK

**Keywords:** NaChBac, NavMs, NavAb, Bacterial sodium channels, Concatenation, Patch clamp, Immunodetection, Western blot

## Abstract

**Background:**

Bacterial sodium channels are important models for understanding ion permeation and selectivity. However, their homotetrameric structure limits their use as models for understanding the more complex eukaryotic voltage-gated sodium channels (which have a pseudo-heterotetrameric structure formed from an oligomer composed of four domains). To bridge this gap we attempted to synthesise oligomers made from four covalently linked bacterial sodium channel monomers and thus resembling their eukaryotic counterparts.

**Results:**

Western blot analyses revealed NaChBac oligomers to be inherently unstable whereas intact expression of NavMs oligomers was possible. Immunodectection using confocal microscopy and electrophysiological characterisation of NavMs tetramers confirmed plasma membrane localisation and equivalent functionality with wild type NavMs channels when expressed in human embryonic kidney cells.

**Conclusion:**

This study has generated new tools for the investigation of eukaryotic channels. The successful covalent linkage of four bacterial Nav channel monomers should permit the introduction of radial asymmetry into the structure of bacterial Nav channels and enable the known structures of these channels to be used to gain unique insights into structure-function relationships of their eukaryotic counterparts.

**Electronic supplementary material:**

The online version of this article (10.1186/s13628-019-0049-5) contains supplementary material, which is available to authorized users.

## Background

Voltage-gated sodium channels (Na_v_s) play fundamental roles in eukaryotes, including electrical signaling, secretion and synaptic transmission. These roles are highlighted in a wide range of diseases (e.g. periodic paralysis, arrhythmia, and epilepsy) which result from the malfunction of mammalian Na_v_s. Eukaryotic Na_v_s are large multi-subunit complexes [1]. The pore-forming subunit is composed of approximately 2000 amino acid residues organized into four domains, each domain comprising six transmembrane spanning (TMS) segments containing a voltage sensor (TMS S4) and a pore forming region (between TMS S5 and S6). Resolving the atomic structure of these proteins is essential for providing a molecular framework to enable elucidation of their function and disease mechanisms. However, their exceptional size and complexity has proved to be a major challenge. Consequently, there is only one report of resolving atomic-level (3.8 Å) structure for eukaryotic Na_v_s [2] and with the caveat of the resolved channel lacking electrophysiological characterization. This current situation represents a significant gap in our understanding of the structure/function relationships of Na_v_s.

The discovery of bacterial Na_v_s has been important in addressing our lack of structural insight into eukaryotic Na_v_s. Bacterial Na_v_s are simplified homologues of eukaryotic Na_v_s; their sequences are analogous to one domain (i.e. six TMS segments with a voltage sensor and a pore forming region) of a eukaryotic Na_v_ and they form functional channels from homotetrameric assembly [3–8]. Their minimalist structure has enabled their atomic-level structures to be resolved, which together with their electrophysiological characterization and atomic simulations based on the resolved structures, have been pivotal in gaining detailed understanding of ion permeation and gating for Na_v_s [9, 10].

However, there are several limitations of these bacterial models for the understanding eucaryotic Na_v_ channels. Foremost is that (unlike eukaryotic Nav channels) bacterial channels display radial symmetry (a consequence of their homotetrameric structure). Consequently, the bacterial counterparts cannot be used to investigate experimentally the distinct role of the four individual domains of eukaryotic Na_v_ channels and as homotetramers, it is not possible to generate asymmetry in a bacterial Na_v_ channel. For example, Xia et al. [11] constructed a model of Na_v_Rh with the selectivity filter (SF) mutated from the radially-symmetrical glutamate ring (EEEE) to the asymmetric ring of DEKA (to mimic the SF in eukaryotic Na_v_ channels) and through MD simulations of Na^+^ permeation proposed a model to explain Na^+^/K^+^ selectivity in mammalian Na_v_ channels. Currently the predictions of the simulation study cannot be experimentally tested.

In an attempt to address this problem and to gain further insights in to the molecular mechanisms of ion permeation in eukaryotic Na_v_ channels using their bacterial counterparts, we attempted to generate a concatenated bacterial Na_v_ channel in which four monomer subunits are covalently linked to form a single polypeptide (and thus mirroring the structure of their eukaryotic counterparts). It was envisaged that such a structure would enable targeted mutation of individual domains of the concatemer and thus permit experimental testing of bacterial channels exhibiting asymmetry in the pore of the bacterial channels (e.g. Xia et al. study). We report intact expression of NaChBac and NavMs and NavAb concatemers but that stable expression was dependent on the expression system employed. Surprisingly, NaChBac concatemer was inherently unstable. However, NavMs concatemers could be expressed intact in mammalian cells and were amenable to electrophysiological investigation using the patch clamp technique.

## Methods

*Generation of expression vectors.* cDNA constructs encoding NaChBac (GenBank accession number BAB05220) and NavMs (GenBank accession number WP_011712479) bacterial sodium channels were synthesized by EPOCH Life Science (www.epochlifescience.com).

NaChBac#1 tetramer was generated by covalently linking four NaChBac monomers (translation stop codons omitted) using hydrophilic linkers containing 16 amino acids (DTQKETLNFGRSTLEI [12]); unique restriction enzyme sites (*EcoRV, SphI and AfeI*) were incorporated between each monomer and a C-terminal FLAG epitope was engineered immediately upstream of the tetramer stop codon, as illustrated in Additional file 2: Figure S5. NaChBac#1 tetramer was subcloned into the pTracer-CMV (Invitrogen) mammalian expression vector at the *EcoRI/XbaI* sites downstream of the constitutive cytomegalovirus (CMV) promoter. Details for the generation of the trimer, dimer and monomer forms of NaChBac#1 are given in Additional file 1.

NaChBac#2, NavAb and NavMs tetramers were generated by covalently linking four identical monomers (translation stop codons omitted) using poly-glycine and the amino acid sequence corresponding to the bovine NCX1 to generate a 61-amino acid linker (GGGGGGGGGGGGGGGGGGGGSHVDHISAETEMEGEGNETGECTGSYYCKKGVILPIWEDEP [13]); unique restriction enzyme sites (*EcoRI, EcoRV and AfeI*) were incorporated between each monomer/linker and a 3xMyc epitope was engineered immediately upstream of the stop codon. Tetramers were subcloned into pcDNA4/HisMaxC mammalian expression vector (EPOCH Life Science) respectively at the *KpnI/XbaI* sites downstream of CMV promoter and in-frame with the Xpress tag, generating an N-terminal Xpress epitope (Additional file 2: Figure S5E). NaChBac#2 and NavMs tetramers were also subcloned into pTracer-CMV vector downstream of cytomegalovirus (CMV) promoter respectively for electrophysiological analysis. To investigate the expression conditions of NachBac#2 tetramer in yeasts and *E. coli*, it was subcloned into the pYES2 yeast expression vector and the pTBX1 bacterial expression vector at sites of *KpnI/XbaI* and *NdeI/NruI* respectively as described in Additional file 1. Plasmid DNA were amplified by DNA Midiprep Kit (Qiagen).

*Cell culture and transfection*. Chinese hamster ovary (CHO) and human embryonic kidney (HEK293T) cells were maintained in DMEM high glucose with L-glutamine (Lonza) supplemented with 10% Fetal Bovine Serum (Gibco) with addition of 50 U/ml Penicillin and 50 μg/ml Streptomycin (Sigma) in a humidified incubator at 37 °C and 5% CO_2_. To introduce the expression of sodium channel genes, 10 μl of *Trans*IT-LT1 reagent (Mirus) and 5 μg of plasmid DNA were equilibrated separately in 250 μl of UltraMEM Reduced Serum Media (Lonza) for 5 min before combining together and incubating at room temperature for another 20 min. The reagent-plasmid mixture was then added to the seeded cells in the 6-well plate dropwise followed by incubating in the incubator overnight.

*Saccharomyces cerevisiae* strain of W303.1a (*MATa ade2–1 ura3–1 his3–11,15 trp1–1 leu2–3112 can1–100*) was cultured at 30 °C and transformed by lithium acetate method [14]; transformants were selected by growth on synthetic complete media without uracil (SCM-ura; Formedium, UK). Competent *E. coli* strain (Rosetta™ DE3; Novagen) was cultured at 37 °C and transformed by heat shock at 42 °C for 30 s; transformants were selected by growth on lysogeny broth (LB) media containing ampicillin.

*Protein extraction.* Protein extraction from CHO and HEK293T cells was performed 18–24 h after transfection. After washing three times with cold PBS buffer containing PierceTM Protease Inhibitor (Thermo Scientific), cells were lysed with RIPA buffer (Sigma) plus phenylmethylsulfonyl fluoride (PMSF) and protease inhibitor on ice for 10 min. The cell lysate was scrapped and transferred to the pre-cooled Eppendorf tubes for collecting supernatant by centrifugation at 13,000 g for 15 min at 4 °C.

Protein extracted from overnight cultures of *S. cerevisiae* (SCM-ura but with glucose replaced with 2% galactose and 2% raffinose to induce protein expression) was conducted by treating yeasts with 2 M of lithium acetate (LiAc) for 5 min and then 0.4 M of NaOH for 10 min at room temperature. Supernatant was tested after centrifugation at 13,000 g for 15 min at 4 °C. Protein expression was induced in *E. coli* by culturing in LB containing 0.4 mM of isopropyl β-D-1-thiogalactopyranoside (IPTG) for 1 h at 37 °C with shaking at 150 rpm. After washing, bacteria were lysed with Y-PER™ Yeast Protein Extraction Reagent according to manufacturer’s instruction (Thermo Scientific) with addition of proteinase inhibitor for 20 min at room temperature. Supernatant after centrifugation at 13,000 g for 15 min was retained for analysis.

*Western blotting*. Proteins were separated using 10% resolving sodium lauryl sulfate polyacrylamide gel electrophoresis (SDS-PAGE) (unless otherwise stated) and transferred to the Hybond™-P PVDF membranes (GE Healthcare Amersham) before blocking with 5% milk. The blots were subsequently treated with primary mouse antibody according to manufacturer’s instruction (Thermo Scientific: Anti-c-Myc monoclonal antibody, MA1–21316; Anti-Xpress monoclonal antibody, R910–25; Sigma-Aldrich: monoclonal ANTI-FLAG® M2 antibody, F1804) at 4 °C overnight followed by secondary antibody (rabbit anti-mouse HRP; Abcam, Ab6728) for 1 h at room temperature. Blots were washed 4 times with 1× phosphate buffered saline (PBS) containing 0.1% tween20 buffer for 5 min after the incubation with either the primary or secondary antibody. Signals were developed by PierceTM ECL substrates (Thermo Scientific) and imaged by ChemiDoc™ (BioRad).

*Electrophysiology*. Whole-cell patch clamp recordings were acquired with Axopatch 200 series amplifiers (Molecular Devices, Sunnyvale, USA). Signals were digitized using Digidata1322 (Molecular Devices, Sunnyvale, USA). Data were filtered at 1 or 2 kHz. All the experiments were performed at 20 °C. Patch pipettes were produced by a pipette puller (model 730, KOPF instrument, USA) from KIMAX melting point capillary tubes (34500–99; Kimble Company, USA). Pipettes had resistances between 2 and 4 MΩ after filling with intracellular solution. Shanks of the pipettes tip were coated with bee’s wax to reduce pipette capacitance. For investigation of the bacterial Nav concatemers, the pipette solution was (in mM) 110 Cs-MetSO3, 20 NaCl, 2 MgCl2, 5 EGTA, 10 HEPES and 2 NaOH (pH 7.2, adjusted with 2 mM NaOH) and the bath solution was (in mM) 140 Na-MetSO3, 2 CaCl2, 10 HEPES, 2 MgCl2, 2 NaOH to pH 7.4 (adjusted with 2 mM NaOH). Data collection was initiated 3 mins after obtaining whole cell configuration to ensure complete equilibration of the pipette solution and cytosol. The bath solution was grounded using a 3 M KCl agar bridge; liquid junction potential determined experimentally [15] agreed with that calculated (using JPCalc program, Clampex, Axon Instruments, Inc.) and was negligible. Results were analyzed using Clampfit 10.1 software (Molecular Devices) and OriginPro8 (OriginLab Corporation). Pooled data are presented as means ± SEM (*n*), where *n* is the number of independent experiments.

## Results and discussion

NaChBac was chosen to be concatenated based on previous reports of successful concatenation [13, 16]. A cDNA was synthesized to form a coding sequence composed of four NaChBac genes concatenated into one open reading frame (Additional file 2: Figure S5). This synthetic gene (referred to here after as NaChBac#1 tetramer) was designed to encode a tetrameric oligomer containing four identical domains corresponding to NaChBac channels (translational stop codon removed) tethered together using a 16 amino acid hydrophilic linker derived from *Xenopus* γ-globin gene (which has been used in the successful expression of K^+^ channel oligomers in mammalian cells [12]). Restriction sites were strategically placed to enable the extraction of individual monomers in the generation of both dimer and trimer constructs (Additional file 2: Figure S5). A C-terminal FLAG epitope was added to enable immunological detection. The DNA constructs were subcloned into mammalian expression vectors for expression in CHO and HEK293T cells for electrophysiological analysis (pTracer-CMV2) and for immunodetection (pIRESneo). It was envisaged that the transfection of cells with the tetramer construct would co-opt the cells biosynthetic machinery into creating a functional Nav channel formed from a single polypeptide and thus with a pre-determined monomer composition.

Immuno-detection of the FLAG epitope and Western blot analyses revealed NaChBac monomer with an approximate (expected [17]) size of 30 kDa (Additional file 2: Figure S6). A protein corresponding to approximately 130 kDa was expected for the intact expression of the NaChBac#1 tetramer; however, only smaller (faint) bands running at approximately 15 kDa (Additional file 2: Figure S6A and B) were detected in both CHO and HEK cells. The size of the degradation products detected in Additional file 2: Figure S6 is consistent with both poor expression and cleavage of the concatemer channels corresponding to a site in the S5 TMS region in the C-terminal domain. A similar pattern of degradation was observed for dimer and trimer constructs (Additional file 2: Figure S6A and B). Immunostaining of transfected CHO cells expressing NaChBac#1 monomer and tetramer and confocal microscopy (Additional file 1 section 3 and Additional file 2: Figure S7) were indistinguishable and showed the FLAG epitope at the plasma membrane of cells. This indicated degradation fragments of the NaChBac oligomers to be present in the plasma membrane and raised the possibility that these “fragments” could interact to form functional channel [18]. To test this possibility, the patch clamp technique was employed to record whole cell plasma membrane ion channel activity in CHO cells expressing NaChBac#1 oligomer proteins. Expression of the NaChBac#1 oligomers produced whole cell currents with similar kinetic properties to that exhibited by cells expressing the NaChBac monomer (Additional file 2: Figure S6C). Thus, oligomer degradation appears to generate lower-order by-products, which associate to form functional channels. Consistent with this, cells expressing NaChBac#1 trimer (which would not be expected to form independent functional channels if intact [19]) also exhibited NaChBac-like whole cell currents (Additional file 2: Figure S6C). Formation of functional channels following degradation of concatenated ATP-gated P2X channels [18] has also been reported.

The failure to express intact NaChBac concatemers in CHO and HEK cells was surprising. The 16 amino acid linker used to generate NaChBac#1 oligomers has previously shown to stably concatenate K^+^ channel monomers [12]; however, a minimum length of linker has also been reported for successful concatenation of GABA receptor subunits [20]. To address the possibility that the linker was too short and to investigate the possibility that stable concatentation depended on the choice of expression system and/or the choice of bacterial Na_v_, alternative concatemers of NaChBac, Na_v_Ms and Na_v_Ab were investigated as detailed below.

### Generation and detection of NaChBac#2, NavMs and NavAb oligomers

A second NaChBac tetramer was generated (referred to here after as NaChBac#2) using a longer (61 amino acid) peptide linker composed of 20x glycine residues and a 41 amino acid linker corresponding to a partial sequence from bovine NCX1. A C-terminal 3xMyc epitope was included to enable immunodetection (Additional file 1). This linker was chosen based on its use in a previous report in which intact NaChBac tetramers were reported in HEK293T cells [13], though a 20× polyglycine linker was also reported for the generation of NaChBac dimers and trimers in CHO [16] and HEK cells [13] respectively. To address the weak expression of degradation products observed in the expression of NaChBac#1 (Additional file 2: Figure S6), the pcDNA4 expression vector was employed for immunodetection. This vector enables the inclusion of an N-terminal Xpress epitope and drives strong expression levels in mammalian cells due to the presence of a QBI SP163 element (a strong translational enhancer aimed at improving expression and detection levels).

**Fig. 1:**
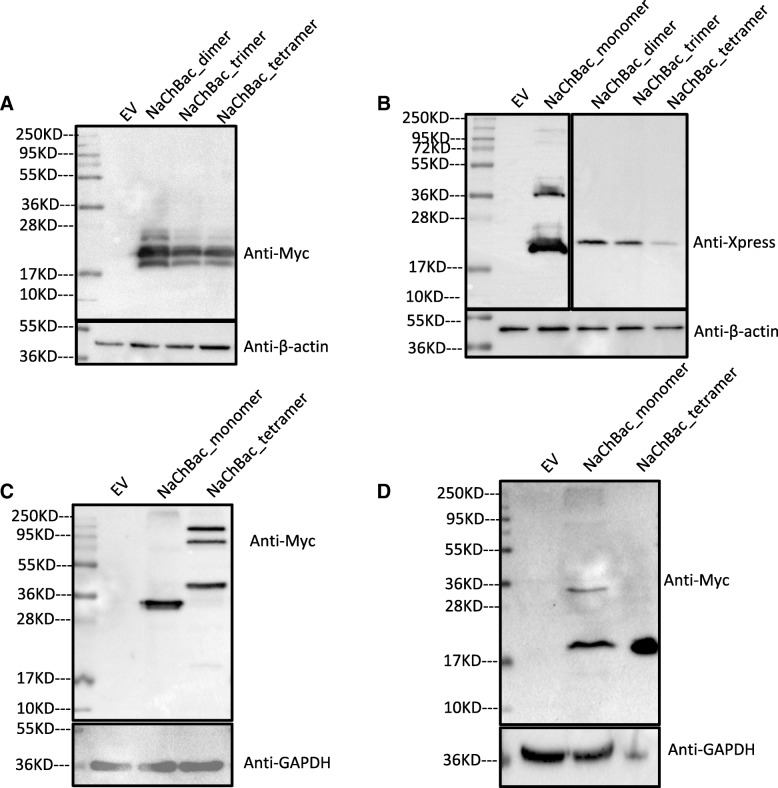
Western blot analyses of total cell protein extracted from (**a**) CHO, (**b**) HEK293T, (**c**) yeast and (**d**) bacterial cells expressing NaChBac#2 oligomers. **a** Upper panel shows reaction to anti-Myc antibody; lower panel is loading control and shows reaction to anti-β-actin antibody. Empty vector (EV) control was the plasmid of pCDNA4. **b** Upper panels show reaction to anti-Xpress antibody; lower panel is loading control and shows reaction to anti-β-actin antibody. EV control was pCDNA4. **c** Upper panel shows reaction to anti-Myc antibody; lower panel is loading control and shows reaction to anti-GAPDH antibody. EV control was pYES2. **d** Upper panel shows reaction to anti-Myc antibody; lower panel is loading control and shows reaction to anti-GAPDH antibody. EV control was pTBX1

Surprisingly, degradation of the NaChBac#2 oligomers was also evident after expression in HEK293T and CHO cells (Fig. 1a and b). Note that the degradation products detected using either N-terminal Xpress tag or a C-terminal Myc tag corresponded to approximate cleavage in the S5 domain (equivalent to that seen for NaChBac#1 oligomer). It is also noteworthy that the strong expression of NaChBac monomer using pCDNA4 also suffers degradation (which is not apparent using more modest expression levels driven by pTracer-CMV). This is consistent with NaChBac being inherently unstable. To investigate the expression in non-mammalian cells, NaChBac#2 tetramer was also expressed in yeast (*Saccharomyces cerevisiae*; Fig. 1c) and bacteria (*Escherichiacoli*; Fig. 1d). Similar degradation of NaChBac#2 tetramer was detected in *E. coli* but intact NaChBac#2 tetramer was apparent in yeast albeit with detectable lower grade degradation. These results suggest that NaChBac oligomers are inherently unstable, though the intact expression in a yeast raises the possibility of using *Pichia pastoris* [21] for high-level protein production (which could be used in downstream biochemical and structural investigations of NaChBac oligomers).

The failure to generate intact NaChBac oligomers in CHO and HEK cells is at odds with previous report [13, 16]. However, Zhao et al. [16] employed only electrophysiological characterization (with no reported immunodetection) in the analysis of their NaChBac dimer construct expressed in CHO cells. Although the electrophysiological characterizations and subsequent interpretations by Zhao et al. are reasonable, this approach cannot exclude the possibility of lower grade degradation. Pavlov et al. [13] reported intact oligomer generation (in HEK cells) free from degradation using Western blot analysis (albeit also at lower expression levels to that reported for the monomer). This contrasts with the present study despite the use of the same linker and expression vector in HEK cells. A possible explanation of this discrepancy may lie in Pavlov et al. using a nickel column to isolate N-terminally HIS-tagged NaChBac prior to Western blotting which may have inadvertently selected for intact products.

**Fig. 2:**
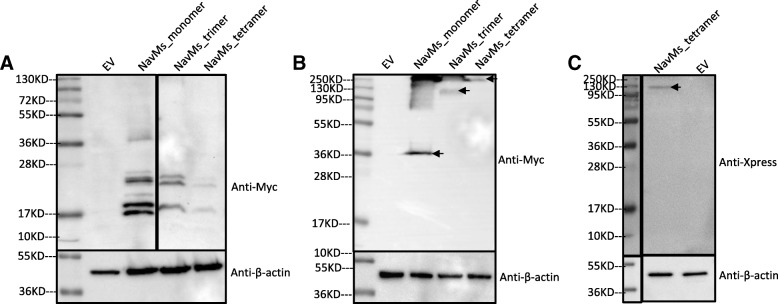
Western blot analyses of total cell protein extracted from (**a**) CHO and (**b** and **c**) HEK293T cells expressing NavMs oligomers. Upper panels show reaction to anti-Myc or Xpress antibody; lower panels are loading control and show reaction to anti-β-actin antibody. EV control was the plasmid of pCDNA4. Arrows indicate expected sizes of intact oligomers

To explore other bacterial voltage-gated sodium channels, equivalent NavAb and NavMs oligomers were generated. NavAb expression in mammalian cells was relatively low (Additional file 2: Figure S8) and difficult to detect consistently; consequently NavAb was not investigated further. Figure 2a shows the detection of Myc epitopes from total protein extracted from CHO cells expressing Na_v_Ms and indicating substantial degradation (note weak expression levels associated with degradation products). However, equivalent expression in HEK cells showed that Na_v_Ms oligomers remained intact with no detectable lower order degradation apparent (Figs. 2b and c). Furthermore, confocal microscopy also showed that the Na_v_Ms tetramer was located on the plasma membrane (Additional file 2: Figure S9). Based on these results, the Na_v_Ms tetramer was taken forward for electrophysiological investigation to establish functionality.

### Electrophysiological properties of concatenated Na_v_Ms oligomers

**Fig. 3:**
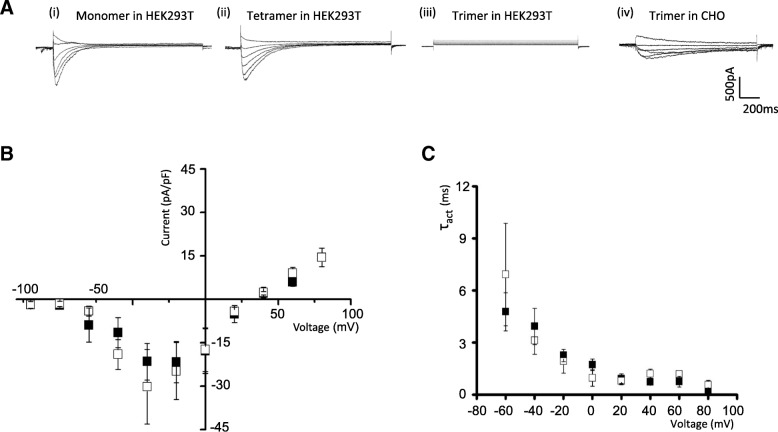
Current density and kinetic properties of NavMs expressed in HEK293T cells. **a** Typical whole cell currents recorded from HEK293 cells transfected to express (i) monomeric, (ii) tetrameric and (iii) trimeric NavMs. (iv) Typical recording from CHO cells expressing trimeric NavMs. Currents were recorded in response to stepping the voltage from 40 mV to − 60 mV in − 20 mV step from a Vhold of − 140 mV. **b** Mean peak current density from HEK293 cells expressing tetrameric (closed squares; *n* = 7), and monomeric (open squares; *n* = 6) NavMs. Error bars represent SEM. **c** Activation kinetic properties (determined from fitting an exponential power function). Currents result from Vhold of − 140 mV). Error bars represent SEM

Figure 3a shows typical whole cell currents from cells expressing monomeric, trimeric and tetrameric forms of Na_v_Ms. Notably, Na_v_Ms-like whole cell currents were detected in HEK cells expressing Na_v_Ms monomer and tetramer but not in cells expressing the trimeric form. This contrasted with Na_v_Ms trimer expression in CHO cells (which showed degradation of NavMs; Fig. 2a) in which whole cell currents consistent with the NavMs oligomer degradation are detectable (Fig. 3a (iv)). The absence of NavMs-mediated currents in HEK cells expressing the trimer constructs confirms the integrity of the concatemer approach to control subunit assembly in the formation of NavMs channels and is consistent with the conclusion that subunits from different concatemers do not interact. Mean current density (Fig. 3b) from cells expressing Na_v_Ms monomer (30.20 ± 12.89 pA/pF; *n* = 7) was similar to that in cells expressing the Na_v_Ms tetramer (21.49 ± 6.30 pA/pF; *n* = 6). Furthermore, fitting whole cell current with an exponential function revealed that the activation time constant (τ) was similar in cells expressing the monomer and tetramer (Fig. 3c) and is similar to that previously reported for Na_v_Ms [22]. Taken together these results show that the NavMs oligomer is intact and exhibits equivalent electrophysiological characteristics to those for the Na_v_Ms monomer.

## Conclusions

The covalent linkage of four bacterial Nav channel monomers resembles the macroscopic structure of their eukaryotic counterparts, which should enable the introduction of radial asymmetry into the structure of bacterial Nav channels. We have created a new tool for the investigation of Na^+^ channels that will enable the physical construction and electrophysiological investigation of bacterial channels (with atomic-resolution structure) exhibiting a SF, for example, composed of an amino acid motif (DEKA) typical of eukaryotic Nav channels. For instance, Flood et al. [24] recently simulated Na^+^ permeation in a simulation model of the human Nav1.2 channel constructed by grafting residues of its selectivity filter and external vestibule region onto a bacterial channel with atomic-resolution structure. Their simulations captured a Na^+^ knock on conduction mechanism in which the DEKA ring lysine (in its protonated form) was seen to form a stable complex with carboxylates and Na^+^. In contrast and in the presence of K^+^, the K^+^-lysine-carboxylate complex is non-existent resulting in the lysine acting as an electrostatic plug blocking K^+^ permeation. The finding that the NavMs bacterial channel can be stably concatenated opens up the possibility of experimentally testing these predictions/modelling results by physically constructing a bacterial channel chimera in which the human Nav1.2 selectivity and vestibule region is grafted onto the NavMs concatemer. A further use of the concatemer is in the testing of ion permeation models which propose that the charge (Q_f_) associated with the amino acid residues forming the SF are the principal factor underlying ion selectivity [23]. In these studies, the selectivity filter of the concatamer can be mutated with the substitution of additional D or E residues to change the value of Q_f_ in steps of *-1e* (as opposed to steps of *-4e* when using the monomer) and thus provide a more detailed investigation of the role of Q_f_ enabling model development.

## Additional files

Additional file 1: Additional information on the construction of bacterial channel concatemers, immunolocalization methodology and description of results. **Figure S1.**, **Figure S2.**, **Figure S3.** and **Figure S4.** which show the DNA sequences of the channel concatemers. (DOCX 30 kb)

Additional file 2: **Table S1.** Listing the primers used for construction of the channel concatemers and **Figure S5**, **Figure S6**, **Figure S7**, **Figure S8** and **Figure S9** which show additional Western blot (and electrophysiological) analyses of channel expression and immunolocalization confocal images of channel constructs. All supplementary figures are referred to in the main text. (PPTX 7.36 mb)

## Abbreviations

CMV: Cytomegalovirus
ITPG: Isopropyl ß-D-1-thiogalactopyranoside
LB: Lysogeny broth
LiAC: Lithium acetate
Navs: Voltage gated sodium channels
PBS: Phosphate buffer saline
PMSF: Phenylmethylsulfonyl 19louride
SCM: Synthetic complete media
SDS PAGE: Sodium lauryl sulfate polyacrylamide gel electrophoresis
TMS: Transmembrane spanning domains
