# Supplementary material for: Covalent linkage of bacterial voltage-gated sodium channels
Source: BMC Biophys. 2019 Apr 27;12:1. doi: 10.1186/s13628-019-0049-5 (PMC6487023; doi:10.1186/s13628-019-0049-5)
Supplement: Supplementary file 1 — Additional information on the construction of bacterial channel concatemers, immunolocalization methodology and description of results. Figure S1., Figure S2., Figure S3. and Figure S4. which show the DNA sequences of the channel concatemers. (DOCX 30 kb) [file 13628_2019_49_MOESM1_ESM.docx]

**Supplementary Information**

**Covalent linkage of bacterial voltage-gated sodium channels**

*Authors*: Huaping Sun, Zeyu Zheng, Olena Federenko, Stephen K. Roberts*

*Division of Biomedical and Life Sciences, Faculty of Health and Medicine, Lancaster University, Lancaster, UK*

*^*^ Corresponding author:*

[s.k.roberts@lancaster.ac.uk](https://exchange2010.lancs.ac.uk/owa/redir.aspx?C=f_nz9aFCOmHmkmFiPk_Gqfcw1dTg0ktteSVST5AG5RcAuuIFRDbUCA..&URL=mailto%3as.k.roberts%40lancaster.ac.uk)

Division of Biomedical and Life Sciences

Faculty of Health and Medicine

Lancaster University

LA1 4YG, UK

Tel: +44 (0)1524 593145

1. **Generation of NaChBac#1 oligomers.**

*Materials and Methods*. NaChBac#1 tetramer (Figure S1) was initially subcloned into pBluescript at the *EcoRV* site (EPOCH Life Science) prior to subcloning into mammalian cell expression vectors of pTracer-CMV2 (Invitrogen) and pIRESneo, downstream of the constitutive cytomegalovirus (CMV) promoter. Unless otherwise stated, all ligations of DNA fragments were performed using T4 DNA ligase (Promega, USA). pTracer-CMV2 constructs were primarily used for electrophysiological analysis (this vector contains GFP for identification of transfected cells) and pIRESneo was primarily used for immunodetection of NaChBac expression.

The *EcoRI/XbaI* fragment encoding the NaChBac tetramer was subcloned into *EcoRI/XbaI* digested pTracer-CMV. For expression using pIRESneo, the tetramer was released from pBluescript by digestion with *XbaI* (the DNA fragment was subsequently ‘blunted’ using DNA polymerase I (Klenow) fragment; NEB) followed by digestion with *EcoRI.* pIRESneo was linearized with *BamHI* and blunted before subsequent digestion with *EcoRI*; subsequent ligation of the tetramer into pIRESneo resulted in regeneration of the *BamHI* site downstream of the ORF stop codon.

NaChBac monomer fragment was generated by PCR amplification of *EcoRI/EcoRV* fragment (Figure S5) using Q5 high fidelity DNA polymerase (NEB) with primers of NaChBac5_EcoRI and NaChBac3_EcoRV (removing the linker and introducing a translation stop codon immediately upstream of the *EcoRV* site). *EcoRI/EcoRV* digestion of the amplicon permitted ligation into pTracer-CMV2; a C-terminal FLAG epitope was subsequently added using Q5® Site-Directed Mutagenesis Kit (NEB) and primers NaCh_add_FLAG_F and NaCh_add_FLAG_R. Primers NaChBac5_EcoRI and Na3_FLAG_BamHI were used to replace the *EcoRV* site with a *BamHI* site (and introduce a FLAG epitope and a translation stop codon immediately upstream of the *BamHI* site); *EcoRI/BamHI* digestion of the amplicon permitted ligation into pIRESneo. The NaChBac dimer was generated by digestion of the tetramer in pTracer-CMV2 with *EcoRV/AfeI* and religation of the pTracer-CMV2 fragment using T4 ligase. The NaChBac trimer was generated by digestion of the tetramer in pTracer-CMV2 with *SphI/AfeI* and PCR amplification of *SphI/AfeI* fragment (Q5 HF polymerase, NEB) using primers TrimerF1 and TrimerR1 (removing the linker sequence and generating a FLAG epitope and two STOP codons upstream of *AfeI*); a Gibson Assembly reaction (NEB) was used to subclone the amplicon back into *SphI/AfeI* digested tetramer in pTracer-CMV2. This product was subsequently digested with *AfeI/XbaI* to remove domain IV and the remaining pTracer-CMV2 fragment was blunted and re-ligated using Quick Blunting and Quick Religation kit (NEB). All clones were sequenced to check for correct construction and to ensure that no unwanted PCR induced mutations had been introduced. DNA for transfection of cells was prepared using Midiprep Plasmid Kit (Qiagen). Primer sequences are given in Table S1 and concatenated NaChBac#1 constructions are summarized in Figure S5A – D.

1. **Generation of NaChBac#2, NavMS and NavAb oligomers.**

*Materials and Methods*. NaChBac#2, NavMs and NavAb tetramers (see Figures S2, S3 and S4) were initially subcloned at the *KpnI/XbaI* site in pcDNA4/HisMaxC plasmid. The *KpnI/XbaI* fragment of NavMs tetramer was digested from pcDNA4/HisMaxC plasmid and subcloned into *KpnI/XbaI* digested pTracer-CMV2. Unless otherwise stated, all ligations of DNA fragments were performed using Quick Ligation™ Kit (NEB). The NavMs monomer was generated by PCR amplification (Q5 high fidelity DNA polymerase; NEB) using the *AfeI/XbaI* NavMs tetramer fragment as template DNA and primers NavMs_EcoRI_F (introducing an EcoRI site and a kozak sequence upstream of the start codon) and NavMs_XbaI_R; the monomer was subloned into pTracer-CMV2at the *EcoRI/XbaI* sites. The NavMs trimer was generated by digestion of the NavMs tetramer in pTracer-CMV2 with *EcoRV* and *AfeI* (to remove domain III) followed by T4 ligase mediated re-ligation.

For expression of NaChBac#2 tetramer in *Saccharomyces cerevisiae* (W303.1a) the NaChBac#2 tetramer was digested from pcDNA4/HisMaxC using *KpnI* and *XbaI* and subcloned downstream of the GAL promoter in pYES2 yeast expression vector at the *KpnI/XbaI* sites. For expression of NaChBac#2 tetramer in *Escherichia coli* the expression vector, pTBX1, was digested with *NdeI* and *NruI*, amplified by PCR using primers pTXB5_NruI and pTXB3_NdeI and a Gibson Assembly reaction (NEB) was performed with the NaChBac#2 tetramer KpnI/XbaI fragment digested from pTracer-CMV2. Details of primers are given in Table S1.

## Immunolocalisation

*Materials and Methods*. Cell culture media was removed and cells were washed 3 times with 1×PBS buffer (Melford Laboratories) before fixation. Cells were fixed by 3.75% of Paraformaldehyde (Thermo Scientific) for 15 minutes at room temperature. Fixed cells were blocked with 1% BSA (Sigma) at room temperature for 1 hour prior to labeling with antibodies. After blocking, cells were incubated with mouse Anti-Xpress monoclonal antibody (R910-25; Thermo Scientific) at 4 ºC overnight followed by secondary antibody Goat anti-mouse IgGs conjugated Alexa Flour® 647 (ab150119; Abcam) incubated at room temperature for 1 hour. Plasma membrane was detected using Anti-alpha 1 Sodium Potassium ATPase antibody conjugated to Alexa Fluor® 488 (ab197496; Abcam). For sequential use of antibodies, cells were washed with 3 ml of 1×PBS-tween20 (0.1%) three times between applications; the sodium potassium ATPase antibody was always applied after detection for primary epitope. After antibody application, cells were mounted (according to manufacturer’s instructions) with VectaShield (Vector Laboratories) with DAPI. For yeast, cells were cultured in SCM-ura with 2% galactose and 2% raffinose at 30 ºC until OD_600_ was 0.6 and 2.5 M sorbitol was added before fixation. Cells were fixed using 3.75% of Paraformaldehyde (Thermo Scientific) for 2.5 hours at room temperature. Fixed cells were pelleted with 2000rpm and washed with 1 ml of PEM for three times, and then incubated with 1 ml of PEM and 0.5 mg of Zymolyase-20T for 70 minutes at room temperature. Cells were subsequently pelleted and washed with 0.5 ml PEM (100 mM PIPES, 1 mM EGTA, 1 mM MgSO_4,_ pH 6.9) containing 1% trition for 2 minutes followed by washing with 1 ml PEM for three times. Samples were blocked with 0.2 ml PEMBAL (100 mM PIPES, 1 mM EGTA, 1 mM MgCl_2_, 1% BSA, 0.1% NaN_3_ and 100 mM Lysine, pH 6.9) agigated for 30 minutes at room temperature. 20 µl samples were re-suspended in 100 µl of PEMBAL containing 10 µl of mouse c-Myc tag monoclonal antibody (1 in 500) (MA1-21316) and incubated at 4 ºC overnight. After washing with 0.5 ml PEM for 5 minutes and three time, the secondary antibody (1:2000) goat anti-mouse IgGs conjugated to AlexaFlour® 647 (ab150119; Abcam) was incubated at 4 ºC overnight. Cells were mounted (according to manufacturer’s instructions) with one drop of Concanavalin A and VectaShield containing DAPI (Vector Laboratories). Prepared cell slides were examined on a confocal microscope (Zeiss LSM 510 Meta laser scanning confocal) and analyzed with Zen software (Zeiss).

*Results*. Immunostaining of transfected cells was performed to determine the intracellular localisation of the NaChBac protein and the extent of its trafficking to the plasma membrane. Immunodetection of the FLAG epitope in CHO cells expressing NaChBac#1 monomer and tetramer (Figure S7) using confocal microscopy was indistinguishable, illustrating equivalent cellular localization. IgGs (conjugated to AlexaFluor488) specific for the Na^+^-K^+^ ATPase were used as a marker for plasma membrane location (Figure S7). An overlapping alignment was observed between the fluorescent patterns for FLAG and Na^+^/K^+^ ATPase indicating a plasma membrane localization of the FLAG epitope.

**Figure S1.** Nucelotide coding sequence encoding the NaChBac#1 tetramer. Unique restriction sites (colour-coded in lower case and underlined) are *EcoRI, XbaI, EcoRV, SphI*, and AfeI. Kozak sequence immediately upstream of the initiation codon is shown in dark blue and underlined. Nucleotide sequence encoding for the amino acid linker is shown in green. The nucleotide sequence encoding for the pore region is denoted by double underline (nucleotides corresponding to amino acids LESWAS are shown in red). Nucleotides encoding for the FLAG epitope are in **lower case and bold**.

gaattcGCCGCCACCATGAAAATGGAAGCTAGACAGAAACAGAACAGCTTCACTAGTAAAATGCAAAAAATCGTGAATCATCGGGCGTTTACGTTTACGGTGATCGCGTTAATTCTTTTTAATGCCCTTATCGTTGGAATCGAAACCTATCCACGTATTTATGCTGATCATAAATGGTTATTCTATCGAATAGATCTTGTTCTTCTCTGGATCTTCACGATTGAAATTGCGATGCGCTTTTTAGCTTCGAATCCGAAATCAGCTTTTTTTCGAAGTTCATGGAATTGGTTTGATTTCTTAATTGTAGCCGCAGGTCATATATTTGCAGGTGCTCAATTTGTGACGGTTCTCCGTATTTTACGGGTTCTCAGGGTACTACGGGCCATCTCAGTTGTTCCATCGTTGCGCAGGTTAGTTGATGCGTTGGTGATGACGATCCCGGCGTTAGGAAACATCTTAATCTTGATGAGCATTTTCTTCTATATTTTTGCCGTTATCGGGACGATGTTATTTCAGCATGTGTCGCCTGAATATTTCGGTAATTTACAGCTTAGTTTGTTAACATTGTTCCAAGTGGTCACGCTAGAGTCATGGGCGAGCGGCGTCATGCGACCAATTTTTGCCGAAGTTCCGTGGTCTTGGCTTTATTTTGTCAGCTTTGTCTTAATCGGTACGTTTATCATCTTTAACTTGTTTATCGGTGTAATCGTCAATAACGTTGAAAAAGCAGAGTTAACGGACAATGAGGAAGATGGTGAAGCCGATGGGTTAAAACAAGAAATCTCAGCCTTAAGAAAAGACGTAGCCGAGCTAAAAAGCTTGCTTAAACAATCGAAAGACACCCAGAAGGAGACCCTGAACTTCGGCAGGTCCACCCTGGAGATCgatatcATGAAAATGGAAGCTAGACAGAAACAGAACAGCTTCACTAGTAAAATGCAAAAAATCGTGAATCATCGGGCGTTTACGTTTACGGTGATCGCGTTAATTCTTTTTAATGCCCTTATCGTTGGAATCGAAACCTATCCACGTATTTATGCTGATCATAAATGGTTATTCTATCGAATAGATCTTGTTCTTCTCTGGATCTTCACGATTGAAATTGCGATGCGCTTTTTAGCTTCGAATCCGAAATCAGCTTTTTTTCGAAGTTCATGGAATTGGTTTGATTTCTTAATTGTAGCCGCAGGTCATATATTTGCAGGTGCTCAATTTGTGACGGTTCTCCGTATTTTACGGGTTCTCAGGGTACTACGGGCCATCTCAGTTGTTCCATCGTTGCGCAGGTTAGTTGATGCGTTGGTGATGACGATCCCGGCGTTAGGAAACATCTTAATCTTGATGAGCATTTTCTTCTATATTTTTGCCGTTATCGGGACGATGTTATTTCAGCATGTGTCGCCTGAATATTTCGGTAATTTACAGCTTAGTTTGTTAACATTGTTtCAgGTtGTtACtCTtGAaagcTGGGCttcaGGtGTtATGaggCCcATTTTTGCCGAAGTTCCGTGGTCTTGGCTTTATTTTGTCAGCTTTGTCTTAATCGGTACGTTTATCATCTTTAACTTGTTTATCGGTGTAATCGTCAATAACGTTGAAAAAGCAGAGTTAACGGACAATGAGGAAGATGGTGAAGCCGATGGGTTAAAACAAGAAATCTCAGCCTTAAGAAAAGACGTAGCCGAGCTAAAAAGCTTGCTTAAACAATCGAAAGACACCCAGAAGGAGACCCTGAACTTCGGCAGGTCCACCCTGGAGATCgcatgcATGAAAATGGAAGCTAGACAGAAACAGAACAGCTTCACTAGTAAAATGCAAAAAATCGTGAATCATCGGGCGTTTACGTTTACGGTGATCGCGTTAATTCTTTTTAATGCCCTTATCGTTGGAATCGAAACCTATCCACGTATTTATGCTGATCATAAATGGTTATTCTATCGAATAGATCTTGTTCTTCTCTGGATCTTCACGATTGAAATTGCGATGCGCTTTTTAGCTTCGAATCCGAAATCAGCTTTTTTTCGAAGTTCATGGAATTGGTTTGATTTCTTAATTGTAGCCGCAGGTCATATATTTGCAGGTGCTCAATTTGTGACGGTTCTCCGTATTTTACGGGTTCTCAGGGTACTACGGGCCATCTCAGTTGTTCCATCGTTGCGCAGGTTAGTTGATGCGTTGGTGATGACGATCCCGGCGTTAGGAAACATCTTAATCTTGATGAGCATTTTCTTCTATATTTTTGCCGTTATCGGGACGATGTTATTTCAGCATGTGTCGCCTGAATATTTCGGTAATTTACAGCTTAGTTTGTTAACATTGTTtCAAGTaGTaACatTgGAGagtTGGGCcAGtGGgGTgATGCGtCCtATTTTTGCCGAAGTTCCGTGGTCTTGGCTTTATTTTGTCAGCTTTGTCTTAATCGGTACGTTTATCATCTTTAACTTGTTTATCGGTGTAATCGTCAATAACGTTGAAAAAGCAGAGTTAACGGACAATGAGGAAGATGGTGAAGCCGATGGGTTAAAACAAGAAATCTCAGCCTTAAGAAAAGACGTAGCCGAGCTAAAAAGCTTGCTTAAACAATCGAAAGACACCCAGAAGGAGACCCTGAACTTCGGCAGGTCCACCCTGGAGATCagcgctATGAAAATGGAAGCTAGACAGAAACAGAACAGCTTCACTAGTAAAATGCAAAAAATCGTGAATCATCGGGCGTTTACGTTTACGGTGATCGCGTTAATTCTTTTTAATGCCCTTATCGTTGGAATCGAAACCTATCCACGTATTTATGCTGATCATAAATGGTTATTCTATCGAATAGATCTTGTTCTTCTCTGGATCTTCACGATTGAAATTGCGATGCGCTTTTTAGCTTCGAATCCGAAATCAGCTTTTTTTCGAAGTTCATGGAATTGGTTTGATTTCTTAATTGTAGCCGCAGGTCATATATTTGCAGGTGCTCAATTTGTGACGGTTCTCCGTATTTTACGGGTTCTCAGGGTACTACGGGCCATCTCAGTTGTTCCATCGTTGCGCAGGTTAGTTGATGCGTTGGTGATGACGATCCCGGCGTTAGGAAACATCTTAATCTTGATGAGCATTTTCTTCTATATTTTTGCCGTTATCGGGACGATGTTATTTCAGCATGTGTCGCCTGAATATTTCGGTAATTTACAGCTTAGTTTGTTAACATTGTTCCAgGTcGTgACctTAGAaTCcTGGGCatcCGGaGTaATGaGACCgATTTTTGCCGAAGTTCCGTGGTCTTGGCTTTATTTTGTCAGCTTTGTCTTAATCGGTACGTTTATCATCTTTAACTTGTTTATCGGTGTAATCGTCAATAACGTTGAAAAAGCAGAGTTAACGGACAATGAGGAAGATGGTGAAGCCGATGGGTTAAAACAAGAAATCTCAGCCTTAAGAAAAGACGTAGCCGAGCTAAAAAGCTTGCTTAAACAATCGAAAGCTGCT**gactacaaggacgacgacgacaag**TGAtctaga

**Figure S2.** Nucelotide coding sequence encoding the NaChBac#2 tetramer

Restriction sites (lower case): (*KpnI* or *Asp718I*) (*Xba1*) (*EcoRV*; *ClaI* also uniquely cuts) (*EcoRI*) (*AfeI*)

**Kozak sequence**:

**Green is the hydrophilic linker**

Underline is the pore region with codon bias **(red is the amino acids corresponding to amino acids LESWAS).** Nucleotides encoding for the 3x Myc epitope are in **bold**.

ggtaccA**GCCGCCACC**ATGAAAATGGAAGCTAGACAGAAACAGAACAGCTTCACTAGTAAAATGCAAAAAATCGTGAATCATCGGGCGTTTACGTTTACGGTGATCGCGTTAATTCTTTTTAATGCCCTTATCGTTGGAATCGAAACCTATCCACGTATTTATGCTGATCATAAATGGTTATTCTATCGAATAGATCTTGTTCTTCTCTGGATCTTCACGATTGAAATTGCGATGCGCTTTTTAGCTTCGAATCCGAAATCAGCTTTTTTTCGAAGTTCATGGAATTGGTTTGATTTCTTAATTGTAGCCGCAGGTCATATATTTGCAGGTGCTCAATTTGTGACGGTTCTCCGTATTTTACGGGTTCTCAGGGTACTACGGGCCATCTCAGTTGTTCCATCGTTGCGCAGGTTAGTTGATGCGTTGGTGATGACGATCCCGGCGTTAGGAAACATCTTAATCTTGATGAGCATTTTCTTCTATATTTTTGCCGTTATCGGGACGATGTTATTTCAGCATGTGTCGCCTGAATATTTCGGTAATTTACAGCTTAGTTTGTTAACATTGTTCCAAGTGGTCACGCTAGAGTCATGGGCGAGCGGCGTCATGCGACCAATTTTTGCCGAAGTTCCGTGGTCTTGGCTTTATTTTGTCAGCTTTGTCTTAATCGGTACGTTTATCATCTTTAACTTGTTTATCGGTGTAATCGTCAATAACGTTGAAAAAGCAGAGTTAACGGACAATGAGGAAGATGGTGAAGCCGATGGGTTAAAACAAGAAATCTCAGCCTTAAGAAAAGACGTAGCCGAGCTAAAAAGCTTGCTTAAACAATCGAAAGGTGGAGGTGGAGGAGGAGGCGGAGGTGGAGGTGGTGGAGGTGGTGGTGGTGGAGGCGGCTCCCACGTGGACCACATCTCCGCCGAGACCGAGATGGAGGGCGAGGGCAACGAGACCGGCGAGTGCACCGGCTCCTACTACTGCAAGAAGGGCGTGATCCTGCCCATCTGGGAGGACGAGCCCgaattcATGAAAATGGAAGCTAGACAGAAACAGAACAGCTTCACTAGTAAAATGCAAAAAATCGTGAATCATCGGGCGTTTACGTTTACGGTGATCGCGTTAATTCTTTTTAATGCCCTTATCGTTGGAATCGAAACCTATCCACGTATTTATGCTGATCATAAATGGTTATTCTATCGAATAGATCTTGTTCTTCTCTGGATCTTCACGATTGAAATTGCGATGCGCTTTTTAGCTTCGAATCCGAAATCAGCTTTTTTTCGAAGTTCATGGAATTGGTTTGATTTCTTAATTGTAGCCGCAGGTCATATATTTGCAGGTGCTCAATTTGTGACGGTTCTCCGTATTTTACGGGTTCTCAGGGTACTACGGGCCATCTCAGTTGTTCCATCGTTGCGCAGGTTAGTTGATGCGTTGGTGATGACGATCCCGGCGTTAGGAAACATCTTAATCTTGATGAGCATTTTCTTCTATATTTTTGCCGTTATCGGGACGATGTTATTTCAGCATGTGTCGCCTGAATATTTCGGTAATTTACAGCTTAGTTTGTTAACATTGTTtCAgGTtGTtACtCTtGAaagcTGGGCttcaGGtGTtATGaggCCcATTTTTGCCGAAGTTCCGTGGTCTTGGCTTTATTTTGTCAGCTTTGTCTTAATCGGTACGTTTATCATCTTTAACTTGTTTATCGGTGTAATCGTCAATAACGTTGAAAAAGCAGAGTTAACGGACAATGAGGAAGATGGTGAAGCCGATGGGTTAAAACAAGAAATCTCAGCCTTAAGAAAAGACGTAGCCGAGCTAAAAAGCTTGCTTAAACAATCGAAAGGTGGAGGTGGAGGAGGAGGCGGAGGTGGAGGTGGTGGAGGTGGTGGTGGTGGAGGCGGCTCCCACGTGGACCACATCTCCGCCGAGACCGAGATGGAGGGCGAGGGCAACGAGACCGGCGAGTGCACCGGCTCCTACTACTGCAAGAAGGGCGTGATCCTGCCCATCTGGGAGGACGAGCCCgatatcATGAAAATGGAAGCTAGACAGAAACAGAACAGCTTCACTAGTAAAATGCAAAAAATCGTGAATCATCGGGCGTTTACGTTTACGGTGATCGCGTTAATTCTTTTTAATGCCCTTATCGTTGGAATCGAAACCTATCCACGTATTTATGCTGATCATAAATGGTTATTCTATCGAATAGATCTTGTTCTTCTCTGGATCTTCACGATTGAAATTGCGATGCGCTTTTTAGCTTCGAATCCGAAATCAGCTTTTTTTCGAAGTTCATGGAATTGGTTTGATTTCTTAATTGTAGCCGCAGGTCATATATTTGCAGGTGCTCAATTTGTGACGGTTCTCCGTATTTTACGGGTTCTCAGGGTACTACGGGCCATCTCAGTTGTTCCATCGTTGCGCAGGTTAGTTGATGCGTTGGTGATGACGATCCCGGCGTTAGGAAACATCTTAATCTTGATGAGCATTTTCTTCTATATTTTTGCCGTTATCGGGACGATGTTATTTCAGCATGTGTCGCCTGAATATTTCGGTAATTTACAGCTTAGTTTGTTAACATTGTTtCAAGTaGTaACatTgGAGagtTGGGCcAGtGGgGTgATGCGtCCtATTTTTGCCGAAGTTCCGTGGTCTTGGCTTTATTTTGTCAGCTTTGTCTTAATCGGTACGTTTATCATCTTTAACTTGTTTATCGGTGTAATCGTCAATAACGTTGAAAAAGCAGAGTTAACGGACAATGAGGAAGATGGTGAAGCCGATGGGTTAAAACAAGAAATCTCAGCCTTAAGAAAAGACGTAGCCGAGCTAAAAAGCTTGCTTAAACAATCGAAAGGTGGAGGTGGAGGAGGAGGCGGAGGTGGAGGTGGTGGAGGTGGTGGTGGTGGAGGCGGCTCCCACGTGGACCACATCTCCGCCGAGACCGAGATGGAGGGCGAGGGCAACGAGACCGGCGAGTGCACCGGCTCCTACTACTGCAAGAAGGGCGTGATCCTGCCCATCTGGGAGGACGAGCCCagcgctATGAAAATGGAAGCTAGACAGAAACAGAACAGCTTCACTAGTAAAATGCAAAAAATCGTGAATCATCGGGCGTTTACGTTTACGGTGATCGCGTTAATTCTTTTTAATGCCCTTATCGTTGGAATCGAAACCTATCCACGTATTTATGCTGATCATAAATGGTTATTCTATCGAATAGATCTTGTTCTTCTCTGGATCTTCACGATTGAAATTGCGATGCGCTTTTTAGCTTCGAATCCGAAATCAGCTTTTTTTCGAAGTTCATGGAATTGGTTTGATTTCTTAATTGTAGCCGCAGGTCATATATTTGCAGGTGCTCAATTTGTGACGGTTCTCCGTATTTTACGGGTTCTCAGGGTACTACGGGCCATCTCAGTTGTTCCATCGTTGCGCAGGTTAGTTGATGCGTTGGTGATGACGATCCCGGCGTTAGGAAACATCTTAATCTTGATGAGCATTTTCTTCTATATTTTTGCCGTTATCGGGACGATGTTATTTCAGCATGTGTCGCCTGAATATTTCGGTAATTTACAGCTTAGTTTGTTAACATTGTTCCAgGTcGTgACctTAGAaTCcTGGGCatcCGGaGTaATGaGACCgATTTTTGCCGAAGTTCCGTGGTCTTGGCTTTATTTTGTCAGCTTTGTCTTAATCGGTACGTTTATCATCTTTAACTTGTTTATCGGTGTAATCGTCAATAACGTTGAAAAAGCAGAGTTAACGGACAATGAGGAAGATGGTGAAGCCGATGGGTTAAAACAAGAAATCTCAGCCTTAAGAAAAGACGTAGCCGAGCTAAAAAGCTTGCTTAAACAATCGAAA**GAGCAGAAGCTGATCTCCGAGGAGGACCTGGAGCAGAAGCTGATCTCCGAGGAGGACCTGGAGCAGAAGCTGATCTCCGAGGAGGACCTG**TAAtctaga

**Figure S3.** Nucelotide coding sequence encoding the NavMs tetramer

Restriction sites: (*KpnI* or *Asp718I*) (*XbaI*) (*EcoRV*) (*EcoRI*) (*AfeI*)

**Kozak sequence**:

**Green is the hydrophilic linker**

*Underline and italics represent alternative codon usage to generate unique priming sites for sequencing.* Nucleotides encoding for the 3x Myc epitope are in **lower case and bold**.

ggtacca**gccgccacc**atgtcacgcaaaataagagatttaatcgaatccaaacgctttcaaaacgtcatcaccgccattattgtgctcaatggcgctgtgctgggtctgctgaccgatacaaccctatcggcctccagccaaaacctgctggagcgtgtggatcaactttgtctgactatctttattgttgaaatatccctgaaaatatacgcctatggcgtgcgaggctttttccgcagcggctggaatctgtttgattttgtgattgtggccatcgcgcttatgcccgcccagggtagcctatcggtgctgcgaaccttccgtatattccgcgtcatgcggctcgtatcggtcataccaaccatgcgaagagtggtgcaaggcatgctcttggcactgcccggcgtgggatcggtagcggcactgttgacggtggtcttctatattgcggctgtcatggccaccaatctctacggggcaaccttccctgaatggtttggtgatcttagcaagagcctgtacacactatttcaggtgatgaccttagagtcatggtctatgggcattgtgcgtccagtgatgaacgttcatcccaacgcatgggtttttttcatccccttcatcatgctcaccacctttaccgtgctcaacctgtttattggcattattgtagatgccatggccatcaccaaggaacaggaggaagaggccaaaaccggccaccaccaagagcctattagccaaacattgctccatctgggagatcgcctagataggatcgaaaagcagcttgcgcaaaacaacgagct*gctgcaaaggcagcaacctcagaag*aaaggtggaggtggaggaggaggcggaggtggaggtggtggaggtggtggtggtggaggcggctcccacgtggaccacatctccgccgagaccgagatggagggcgagggcaacgagaccggcgagtgcaccggctcctactactgcaagaagggcgtgatcctgcccatctgggaggacgagcccgaattcatgtc*taggaagatccgc*gatttaatcgaatccaaacgctttcaaaacgtcatcaccgccattattgtgctcaatggcgctgtgctgggtctgctgaccgatacaaccctatcggcctccagccaaaacctgctggagcgtgtggatcaactttgtctgactatctttattgttgaaatatccctgaaaatatacgcctatggcgtgcgaggctttttccgcagcggctggaatctgtttgattttgtgattgtggccatcgcgcttatgcccgcccagggtagcctatcggtgctgcgaaccttccgtatattccgcgtcatgcggctcgtatcggtcataccaaccatgcgaagagtggtgcaaggcatgctcttggcactgcccggcgtgggatcggtagcggcactgttgacggtggtcttctatattgcggctgtcatggccaccaatctctacggggcaaccttccctgaatggtttggtgatcttagcaagagcctgtacacactatttcaggtgatgaccttagagtcatggtctatgggcattgtgcgtccagtgatgaacgttcatcccaacgcatgggtttttttcatccccttcatcatgctcaccacctttaccgtgctcaacctgtttattggcattattgtagatgccatggccatcaccaaggaacaggaggaagaggccaaaaccggccaccaccaagagcctattagccaaacattgctccatctgggagatcgcctagataggatcgaaaagcagct*ggctcagaacaatgaactcctg*caacgacaacagccgcaaaaaaaaggtggaggtggaggaggaggcggaggtggaggtggtggaggtggtggtggtggaggcggctcccacgtggaccacatctccgccgagaccgagatggagggcgagggcaacgagaccggcgagtgcaccggctcctactactgcaagaagggcgtgatcctgcccatctgggaggacgagcccgatatcatgtcacgcaaaataagagatttaatcgaatccaaaaggttccagaatgtgattaccgccattattgtgctcaatggcgctgtgctgggtctgctgaccgatacaaccctatcggcctccagccaaaacctgctggagcgtgtggatcaactttgtctgactatctttattgttgaaatatccctgaaaatatacgcctatggcgtgcgaggctttttccgcagcggctggaatctgtttgattttgtgattgtggccatcgcgcttatgcccgcccagggtagcctatcggtgctgcgaaccttccgtatattccgcgtcatgcggctcgtatcggtcataccaaccatgcgaagagtggtgcaaggcatgctcttggcactgcccggcgtgggatcggtagcggcactgttgacggtggtcttctatattgcggctgtcatggccaccaatctctacggggcaaccttccctgaatggtttggtgatcttagcaagagcctgtacacactatttcaggtgatgaccttagagtcatggtctatgggcattgtgcgtccagtgatgaacgttcatcccaacgcatgggtttttttcatccccttcatcatgctcaccacctttaccgtgctcaacctgtttattggcattattgtagatgccatggccatcaccaaggaacaggaggaagaggccaaaaccggccaccaccaagagcctattagccaaaca*ctcctgcacctcggtgacaga*ctagataggatcgaaaagcagcttgcgcaaaacaacgagctcttacaacgacaacagccgcaaaaaaaaggtggaggtggaggaggaggcggaggtggaggtggtggaggtggtggtggtggaggcggctcccacgtggaccacatctccgccgagaccgagatggagggcgagggcaacgagaccggcgagtgcaccggctcctactactgcaagaagggcgtgatcctgcccatctgggaggacgagcccagcgctatgtcacgcaaaataagagatttaatcgaatccaaacgctttcaaaacgtcatcaccgccattattgtgctcaatggcgctgtgctgggtctgctgaccgatacaaccctatcggcctccagccaaaacctgctggagcgtgtggatcaactttgtctgactatctttattgttgaaatatccctgaaaatatacgcctatggcgtgcgaggctttttccgcagcggctggaatctgtttgattttgtgattgtggccatcgcgcttatgcccgcccagggtagcctatcggtgctgcgaaccttccgtatattccgcgtcatgcggctcgtatcggtcataccaaccatgcgaagagtggtgcaaggcatgctcttggcactgcccggcgtgggatcggtagcggcactgttgacggtggtcttctatattgcggctgtcatggccaccaatctctacggggcaaccttccctgaatggtttggtgatcttagcaagagcctgtacacactatttcaggtgatgaccttagagtcatggtctatgggcattgtgcgtccagtgatgaacgttcatcccaacgcatgggtttttttcatccccttcatcatgctcaccacctttaccgtgctcaacctgtttattggcattattgtagatgccatggccatcaccaaggaacaggaggaagaggccaaaaccggccaccaccaagagcctattagccaaacattgctccatctgggagatcgcctagataggatcgaaaagcagcttgcgcaaaacaacgagctcttacaacgacaacagccgcaaaaaaaa**gagcagaagctgatctccgaggaggacctggagcagaagctgatctccgaggaggacctggagcagaagctgatctccgaggaggacctg**taatctaga

**Figure S4.** Nucelotide coding sequence encoding the NavAb tetramer

Restriction sites: (*KpnI* or *Asp718I*) (*XbaI*) (*EcoRV*) (*EcoRI*) (*AfeI*)

**Kozak sequence**:

**Green is the hydrophilic linker**

*Underline and italics represent alternative codon usage to generate unique priming sites for sequencing.* Nucleotides encoding for the 3x Myc epitope are in **lower case and bold**.

ggtaccagccgccaccatgtatcttagaattacaaatattgtagaaagtagctttttcacaaagtttataatttatttaattgtattaaatggaattacaatgggacttgagacttcaaaaacttttatgcaaagttttggagtttatacaacgctatttaatcaaattgttattactatttttactatagagattattttaagaatttatgttcatagaatctcttttttcaaagacccatggagtctatttgattttttcgttgttgctatctctttagttcctacaagttcaggatttgaaatattaagagttttaagagttcttagactatttaggttagttactgctgttcctcaaatgagaaaaatagtttcagcacttattagtgtgattccaggaatgttatctgtaattgctttaatgacactgtttttttatatttttgcaattatggcaacacaactttttggagaaagatttcctgaatggtttggaacattaggtgaatctttttatacactatttcaagttatgactttagaatcttggtcaatgggtattgtcagacctctgatggaagtttatccttatgcttgggttttctttataccttttatatttgtagtaacttttgtaatgataaatttagtggttgctattatagttgatgcaatggctattttaaatcaaaaagaggaacaacatataattgatgaagttcaatctcatgaagataatataaataatgagataataaaattaagagaagagattgtagaacttaaagaattaataaaaactagtttaaaaaacggtggaggtggaggaggaggcggaggtggaggtggtggaggtggtggtggtggaggcggctcccacgtggaccacatctccgccgagaccgagatggagggcgagggcaacgagaccggcgagtgcaccggctcctactactgcaagaagggcgtgatcctgcccatctgggaagatgaacctgaattcatgtatcttagaattacaaatattgtagaaagtagctttttcacaaagtttataatttatttaattgtattaaatggaattacaatgggacttgagacttcaaaaacttttatgcaaagttttggagtttatacaacgctatttaatcaaattgttattactatttttactatagagattattttaagaatttatgttcatagaatctcttttttcaaagacccatggagtctatttgattttttcgttgttgctatctctttagttcctacaagttcaggatttgaaatattaagagttttaagagttcttagactatttaggttagttactgctgttcctcaaatgagaaaaatagtttcagcacttattagtgtgattccaggaatgttatctgtaattgctttaatgacactgtttttttatatttttgcaattatggcaacacaactttttggagaaagatttcctgaatggtttggaacattaggtgaatctttttatacactatttcaagttatgactttagaatcttggtcaatgggtattgtcagacctctgatggaagtttatccttatgcttgggttttctttataccttttatatttgtagtaacttttgtaatgataaatttagtggttgctattatagttgatgcaatggctattttaaatcaaaaagaggaacaacatataattgatgaagttcaatctcatgaagataatataaataatgagataataaaattaagagaagagattgtagaacttaaagaattaataaa*gacctccctgaagaat*ggtggaggtggaggaggaggcggaggtggaggtggtggaggtggtggtggtggaggcggctcccacgtggaccacatctccgccgagaccgagatggagggcgagggcaacgagaccggcgagtgcaccggctcctactactgcaagaagggcgtgatcctgcccatctgggaggacgagcccgatatcatgtatcttagaattacaaatattgtagaaagtagctttttcacaaagtttataatttatttaattgtattaaatggaattacaatgggacttgagacttcaaaaacttttatgcaaagttttggagtttatacaacgctatttaatcaaattgttattactatttttactatagagattattttaagaatttatgttcatagaatctcttttttcaaagacccatggagtctatttgattttttcgttgttgctatctctttagttcctacaagttcaggatttgaaatattaagagttttaagagttcttagactatttaggttagttactgctgttcctcaaatgagaaaaatagtttcagcacttattagtgtgattccaggaatgttatctgtaattgctttaatgacactgtttttttatatttttgcaattatggcaacacaactttttggagaaagatttcctgaatggtttggaacattaggtgaatctttttatacactatttcaagttatgactttagaatcttggtcaatgggtattgtcagacctctgatggaagtttatccttatgcttgggttttctttataccttttatatttgtagtaacttttgtaatgataaatttagtggttgctattatagttgatgcaatggctattttaaatcaaaaagaggaacaacatataattgatgaagttcaatctcatgaagataatataaataatgagataataaaattaagagaagagattgtagaacttaaagaa*ctgatcaagacatctctcaagaa*cggaggaggtggaggaggaggcggaggtggaggtggtggaggtggtggtggtggaggcggctcccacgtggaccacatctccgccgagaccgagatggagggcgagggcaacgagaccggcgagtgcaccggctcctactactgcaagaagggcgtgatcctgcccatctgggaggacgagccaagcgctatgtacctgcgtattacaaatattgtagaaagtagctttttcacaaagtttataatttatttaattgtattaaatggaattacaatgggacttgagacttcaaaaacttttatgcaaagttttggagtttatacaacgctatttaatcaaattgttattactatttttactatagagattattttaagaatttatgttcatagaatctcttttttcaaagacccatggagtctatttgattttttcgttgttgctatctctttagttcctacaagttcaggatttgaaatattaagagttttaagagttcttagactatttaggttagttactgctgttcctcaaatgagaaaaatagtttcagcacttattagtgtgattccaggaatgttatctgtaattgctttaatgacactgtttttttatatttttgcaattatggcaacacaactttttggagaaagatttcctgaatggtttggaacattaggtgaatctttttatacactatttcaagttatgactttagaatcttggtcaatgggtattgtcagacctctgatggaagtttatccttatgcttgggttttctttataccttttatatttgtagtaacttttgtaatgataaatttagtggttgctattatagttgatgcaatggctattttaaatcaaaaagaggaacaacatataattgatgaagttcaatctcatgaagataatataaataatgagataataaaattaagagaagagattgtagaacttaaagaattaataaaaactagc*ttgaag*aac**gagcagaagctgatctccgaggaggacctggagcagaagctgatctccgaggaggacctggagcagaagctgatctccgaggaggacctg**taatctaga
