# Supplementary material for: Covalent linkage of bacterial voltage-gated sodium channels
Source: BMC Biophys. 2019 Apr 27;12:1. doi: 10.1186/s13628-019-0049-5 (PMC6487023; doi:10.1186/s13628-019-0049-5)
Supplement: Supplementary file 2 — Table S1. Listing the primers used for construction of the channel concatemers and Figure S5, Figure S6, Figure S7, Figure S8 and Figure S9 which show additional Western blot (and electrophysiological) analyses of channel expression and immunolocalization confocal images of channel constructs. All supplementary figures are referred to in the main text. (PPTX 7.36 mb) [file 13628_2019_49_MOESM2_ESM.pptx]

## Slide 1
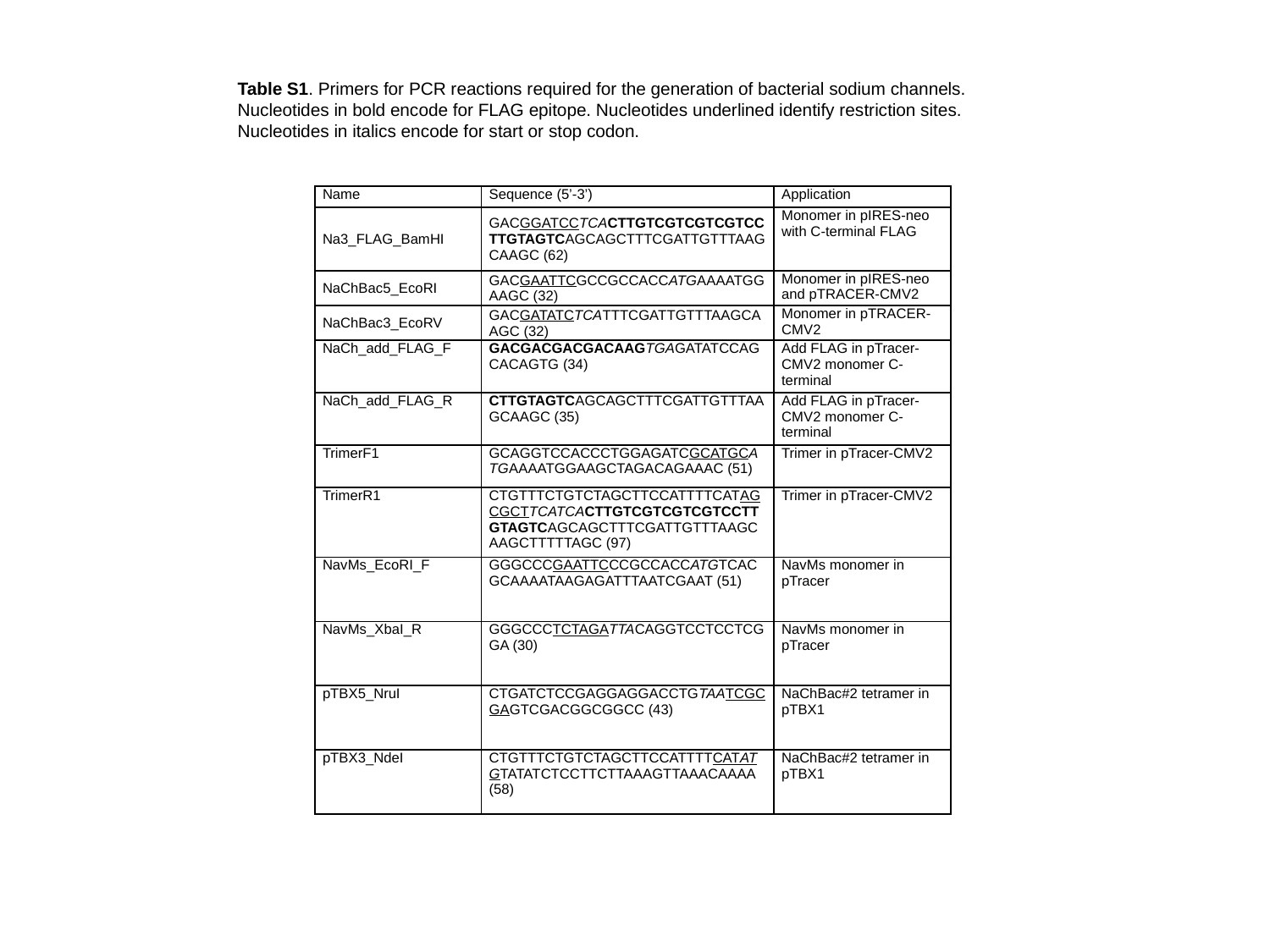

Table S1. Primers for PCR reactions required for the generation of bacterial sodium channels. Nucleotides in bold encode for FLAG epitope. Nucleotides underlined identify restriction sites. Nucleotides in italics encode for start or stop codon.
| Name | Sequence (5’-3’) | Application |
| --- | --- | --- |
| Na3\_FLAG\_BamHI | GACGGATCCTCACTTGTCGTCGTCGTCCTTGTAGTCAGCAGCTTTCGATTGTTTAAGCAAGC (62) | Monomer in pIRES-neo with C-terminal FLAG |
| NaChBac5\_EcoRI | GACGAATTCGCCGCCACCATGAAAATGGAAGC (32) | Monomer in pIRES-neo and pTRACER-CMV2 |
| NaChBac3\_EcoRV | GACGATATCTCATTTCGATTGTTTAAGCAAGC (32) | Monomer in pTRACER-CMV2 |
| NaCh\_add\_FLAG\_F | GACGACGACGACAAGTGAGATATCCAGCACAGTG (34) | Add FLAG in pTracer-CMV2 monomer C-terminal |
| NaCh\_add\_FLAG\_R | CTTGTAGTCAGCAGCTTTCGATTGTTTAAGCAAGC (35) | Add FLAG in pTracer-CMV2 monomer C-terminal |
| TrimerF1 | GCAGGTCCACCCTGGAGATCGCATGCATGAAAATGGAAGCTAGACAGAAAC (51) | Trimer in pTracer-CMV2 |
| TrimerR1 | CTGTTTCTGTCTAGCTTCCATTTTCATAGCGCTTCATCACTTGTCGTCGTCGTCCTTGTAGTCAGCAGCTTTCGATTGTTTAAGCAAGCTTTTTAGC (97) | Trimer in pTracer-CMV2 |
| NavMs\_EcoRI\_F | GGGCCCGAATTCCCGCCACCATGTCACGCAAAATAAGAGATTTAATCGAAT (51) | NavMs monomer in pTracer |
| NavMs\_XbaI\_R | GGGCCCTCTAGATTACAGGTCCTCCTCGGA (30) | NavMs monomer in pTracer |
| pTBX5\_NruI | CTGATCTCCGAGGAGGACCTGTAATCGCGAGTCGACGGCGGCC (43) | NaChBac#2 tetramer in pTBX1 |
| pTBX3\_NdeI | CTGTTTCTGTCTAGCTTCCATTTTCATATGTATATCTCCTTCTTAAAGTTAAACAAAA (58) | NaChBac#2 tetramer in pTBX1 |

## Slide 2
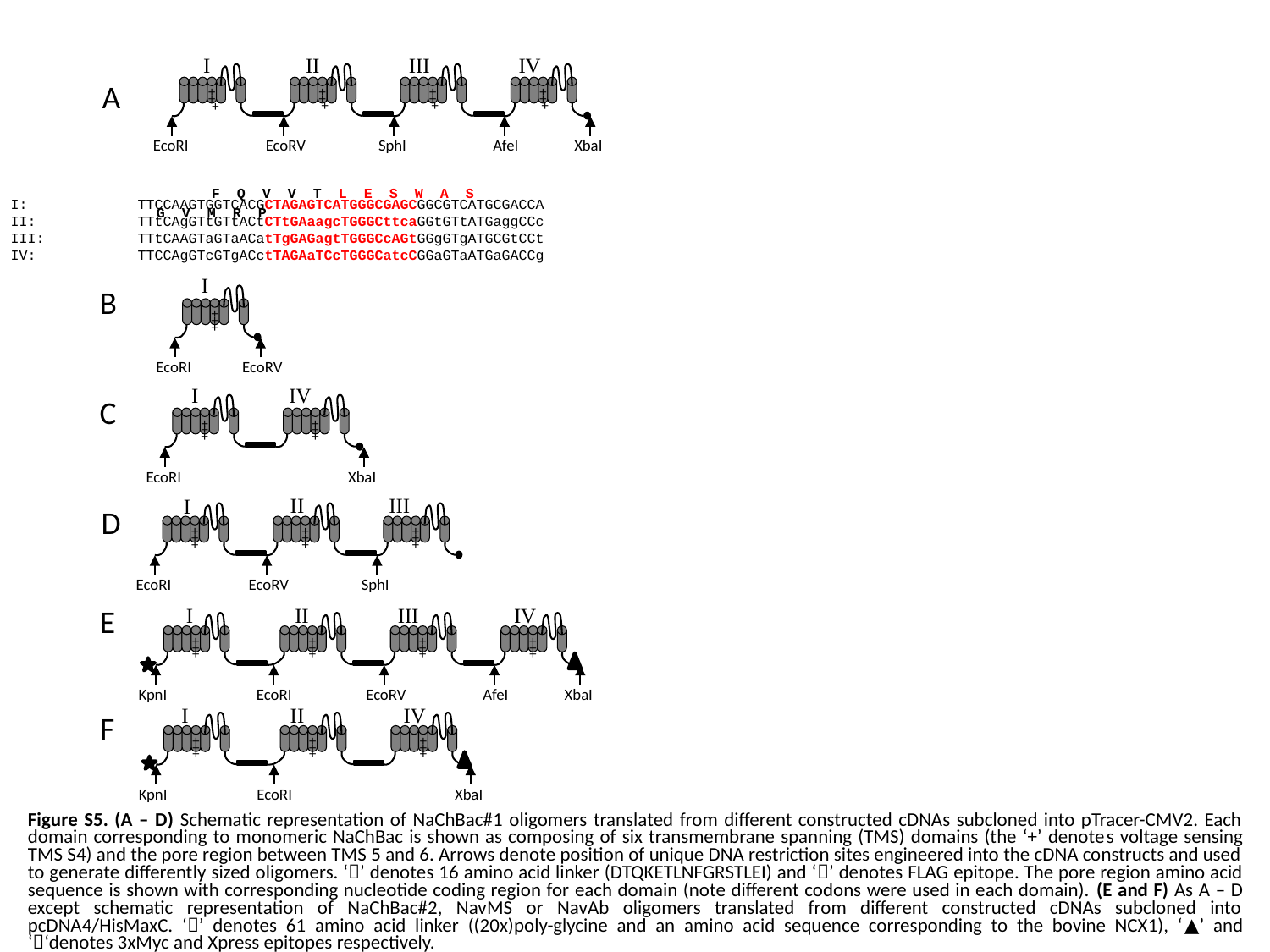

I
II
III
IV
+
+
+
+
+
+
+
+
+
A
+
+
 +
EcoRI
EcoRV
SphI
AfeI
XbaI
F Q V V T L E S W A S G V M R P
I: 	TTCCAAGTGGTCACGCTAGAGTCATGGGCGAGCGGCGTCATGCGACCA
II: 	TTtCAgGTtGTtACtCTtGAaagcTGGGCttcaGGtGTtATGaggCCc
III: 	TTtCAAGTaGTaACatTgGAGagtTGGGCcAGtGGgGTgATGCGtCCt
IV:	TTCCAgGTcGTgACctTAGAaTCcTGGGCatcCGGaGTaATGaGACCg
I
+
+
+
EcoRI
EcoRV
B
I
IV
+
+
+
+
+
+
EcoRI
XbaI
C
II
III
I
+
+
+
+
+
+
+
+
+
EcoRI
EcoRV
SphI
D
E
+
+
+
+
+
+
+
+
+
+
+
+
KpnI
EcoRI
EcoRV
AfeI
XbaI
III
I
II
IV
I
II
IV
F
+
+
+
+
+
+
+
+
+
KpnI
EcoRI
XbaI
Figure S5. (A – D) Schematic representation of NaChBac#1 oligomers translated from different constructed cDNAs subcloned into pTracer-CMV2. Each domain corresponding to monomeric NaChBac is shown as composing of six transmembrane spanning (TMS) domains (the ‘+’ denotes voltage sensing TMS S4) and the pore region between TMS 5 and 6. Arrows denote position of unique DNA restriction sites engineered into the cDNA constructs and used to generate differently sized oligomers. ‘’ denotes 16 amino acid linker (DTQKETLNFGRSTLEI) and ‘’ denotes FLAG epitope. The pore region amino acid sequence is shown with corresponding nucleotide coding region for each domain (note different codons were used in each domain). (E and F) As A – D except schematic representation of NaChBac#2, NavMS or NavAb oligomers translated from different constructed cDNAs subcloned into pcDNA4/HisMaxC. ‘’ denotes 61 amino acid linker ((20x)poly-glycine and an amino acid sequence corresponding to the bovine NCX1), ‘▲’ and ‘‘denotes 3xMyc and Xpress epitopes respectively.

## Slide 3
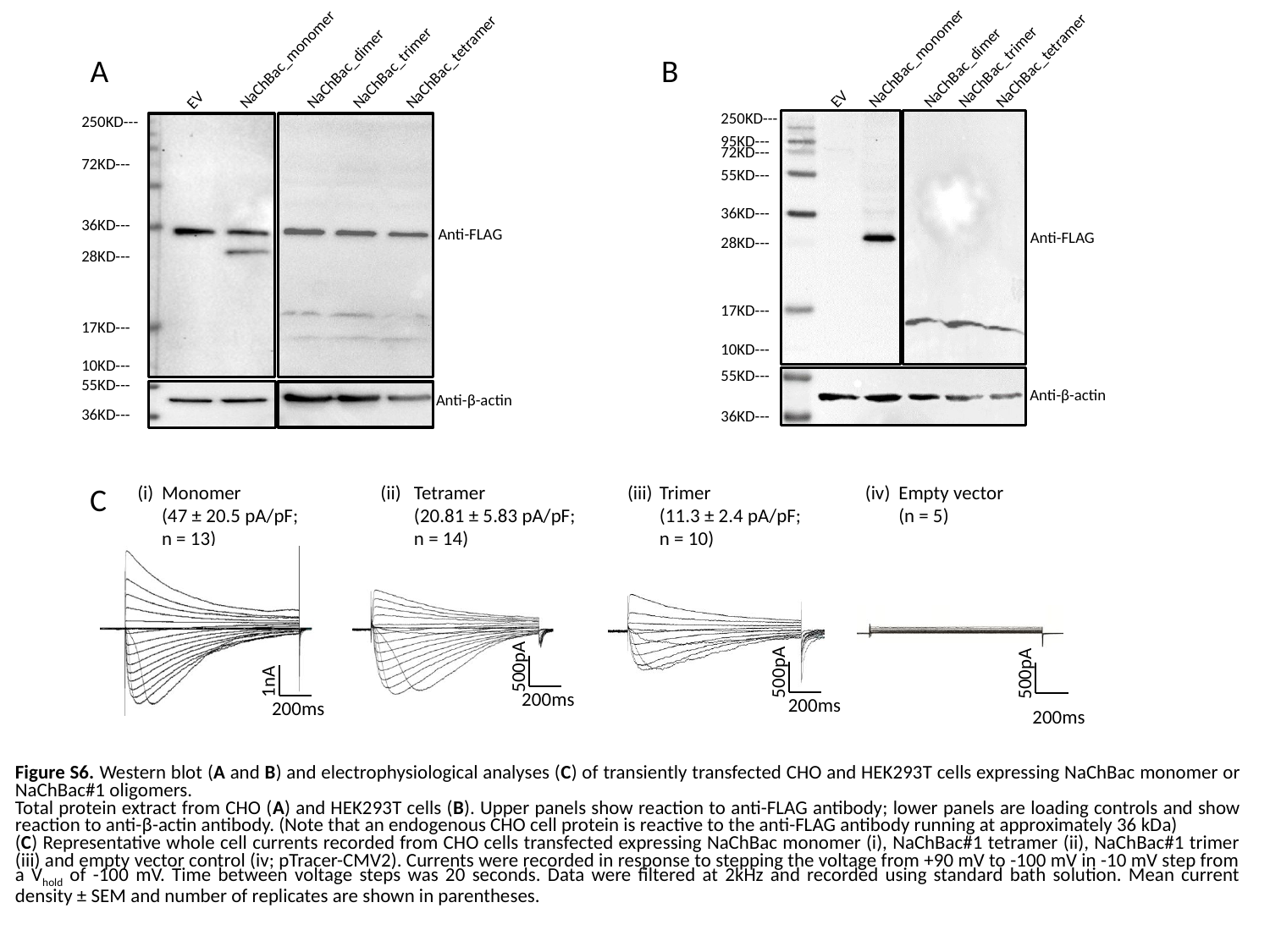

NaChBac_monomer
NaChBac_dimer
NaChBac_trimer
NaChBac_tetramer
EV
250KD---
95KD---
72KD---
55KD---
36KD---
28KD---
17KD---
10KD---
55KD---
Anti-β-actin
36KD---
NaChBac_monomer
NaChBac_dimer
NaChBac_trimer
NaChBac_tetramer
EV
250KD---
72KD---
36KD---
28KD---
17KD---
10KD---
55KD---
Anti-β-actin
36KD---
A
B
Anti-FLAG
Anti-FLAG
Monomer
(47 ± 20.5 pA/pF;
n = 13)
C
(i)
(ii)
Tetramer
(20.81 ± 5.83 pA/pF;
n = 14)
(iii)
Trimer
(11.3 ± 2.4 pA/pF;
n = 10)
(iv)
Empty vector
(n = 5)
 1nA
200ms
500pA
200ms
500pA
200ms
500pA
200ms
Figure S6. Western blot (A and B) and electrophysiological analyses (C) of transiently transfected CHO and HEK293T cells expressing NaChBac monomer or NaChBac#1 oligomers.
Total protein extract from CHO (A) and HEK293T cells (B). Upper panels show reaction to anti-FLAG antibody; lower panels are loading controls and show reaction to anti-β-actin antibody. (Note that an endogenous CHO cell protein is reactive to the anti-FLAG antibody running at approximately 36 kDa)
(C) Representative whole cell currents recorded from CHO cells transfected expressing NaChBac monomer (i), NaChBac#1 tetramer (ii), NaChBac#1 trimer (iii) and empty vector control (iv; pTracer-CMV2). Currents were recorded in response to stepping the voltage from +90 mV to -100 mV in -10 mV step from a Vhold of -100 mV. Time between voltage steps was 20 seconds. Data were filtered at 2kHz and recorded using standard bath solution. Mean current density ± SEM and number of replicates are shown in parentheses.

## Slide 4
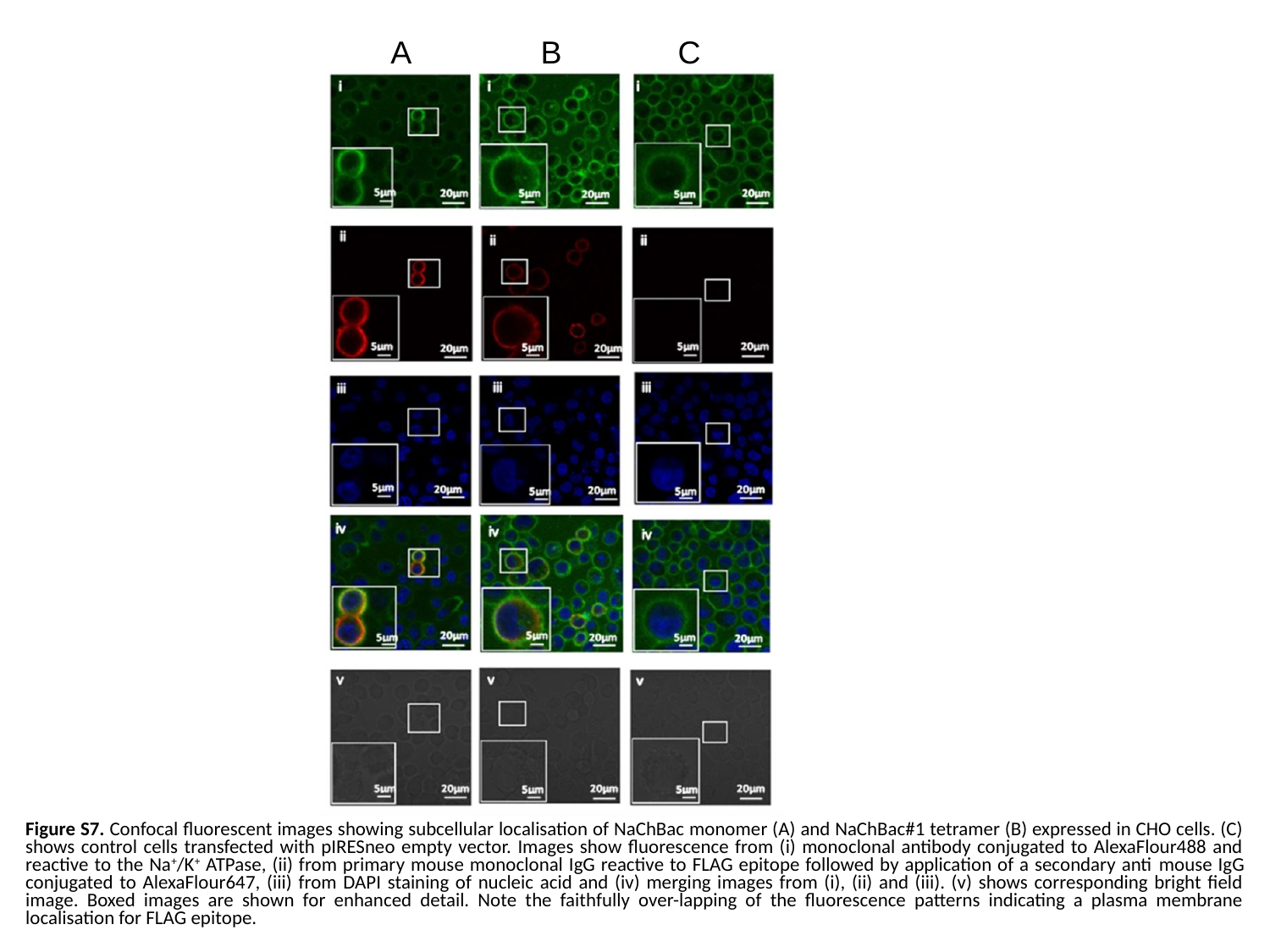

A
B
C
Figure S7. Confocal fluorescent images showing subcellular localisation of NaChBac monomer (A) and NaChBac#1 tetramer (B) expressed in CHO cells. (C) shows control cells transfected with pIRESneo empty vector. Images show fluorescence from (i) monoclonal antibody conjugated to AlexaFlour488 and reactive to the Na+/K+ ATPase, (ii) from primary mouse monoclonal IgG reactive to FLAG epitope followed by application of a secondary anti mouse IgG conjugated to AlexaFlour647, (iii) from DAPI staining of nucleic acid and (iv) merging images from (i), (ii) and (iii). (v) shows corresponding bright field image. Boxed images are shown for enhanced detail. Note the faithfully over-lapping of the fluorescence patterns indicating a plasma membrane localisation for FLAG epitope.

## Slide 5
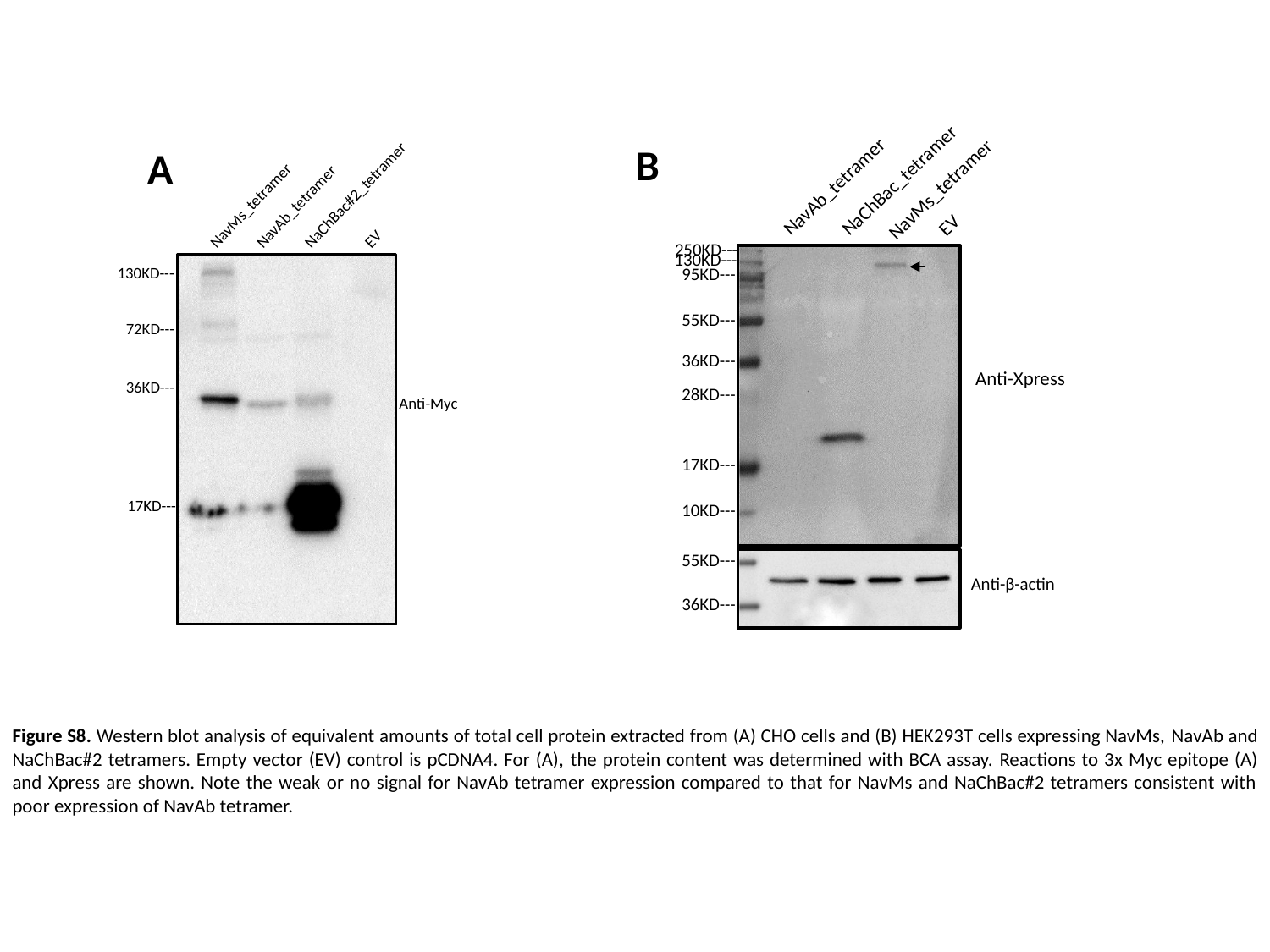

NavMs_tetramer
NavAb_tetramer
NaChBac#2_tetramer
EV
130KD---
72KD---
36KD---
17KD---
Anti-Myc
B
NavAb_tetramer
NaChBac_tetramer
NavMs_tetramer
Anti-Xpress
EV
250KD---
130KD---
95KD---
55KD---
36KD---
28KD---
17KD---
10KD---
55KD---
36KD---
Anti-β-actin
A
Figure S8. Western blot analysis of equivalent amounts of total cell protein extracted from (A) CHO cells and (B) HEK293T cells expressing NavMs, NavAb and NaChBac#2 tetramers. Empty vector (EV) control is pCDNA4. For (A), the protein content was determined with BCA assay. Reactions to 3x Myc epitope (A) and Xpress are shown. Note the weak or no signal for NavAb tetramer expression compared to that for NavMs and NaChBac#2 tetramers consistent with poor expression of NavAb tetramer.

## Slide 6
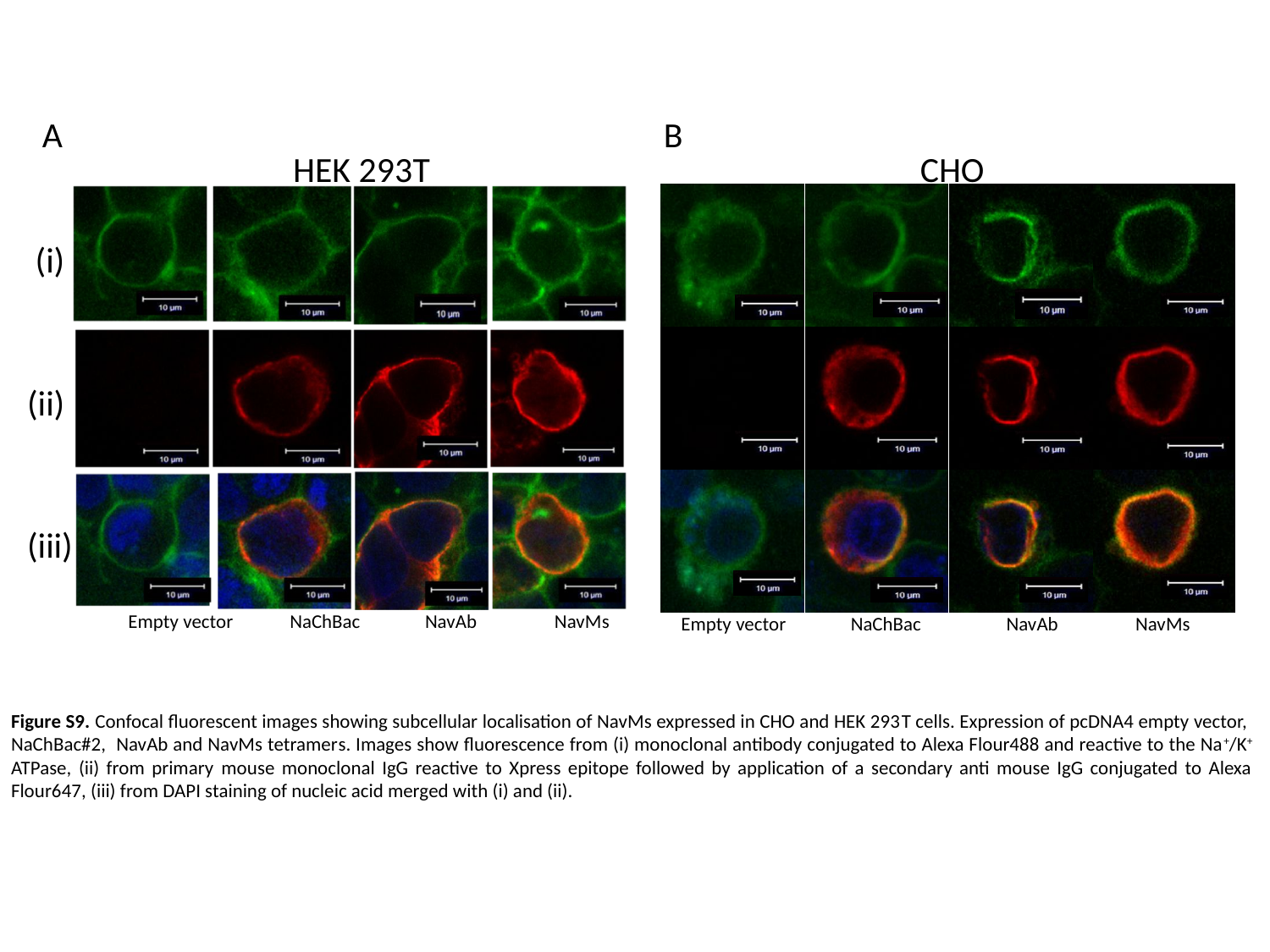

A
B
 HEK 293T
CHO
(i)
(ii)
(iii)
Empty vector
NaChBac
NavAb
NavMs
NaChBac
NavMs
Empty vector
NavAb
Figure S9. Confocal fluorescent images showing subcellular localisation of NavMs expressed in CHO and HEK 293T cells. Expression of pcDNA4 empty vector, NaChBac#2, NavAb and NavMs tetramers. Images show fluorescence from (i) monoclonal antibody conjugated to Alexa Flour488 and reactive to the Na+/K+ ATPase, (ii) from primary mouse monoclonal IgG reactive to Xpress epitope followed by application of a secondary anti mouse IgG conjugated to Alexa Flour647, (iii) from DAPI staining of nucleic acid merged with (i) and (ii).
